# State Minimum Wage Increases As a Potential Policy Lever to Reduce Black–White Disparities in Hypertension

**DOI:** 10.1089/heq.2022.0192

**Published:** 2023-05-23

**Authors:** Brittany L. Brown-Podgorski, Stephanie Doran-Brubaker, Shetal Vohra-Gupta

**Affiliations:** ^1^ Department of Health Policy and Management, School of Public Health, University of Pittsburgh, Pittsburgh, Pennsylvania, USA.; ^2^ Steve Hicks School of Social Work, University of Texas at Austin, Austin, Texas, USA.

**Keywords:** hypertension, racial health disparities, minimum wage, structural racism, policy analysis

## Abstract

**Introduction:**

Black adults are disproportionately burdened by hypertension. Income inequality is associated with elevated risk of hypertension. Minimum wage increases have been explored as a potential policy lever to address the disparate impact of hypertension on this population. However, these increases may have no significant impact on health among Black adults due to structural racism and “diminished gain” of health effects from socioeconomic resources. This study assesses the relationship between state minimum wage increases and Black–White disparities in hypertension.

**Methods:**

We merged state-level minimum wage data with survey data from the Behavioral Risk Factor Surveillance System (2001–2019). Odd survey years included questions about hypertension. Separate difference-in-difference models estimated the odds of hypertension among Black and White adults in states with and without minimum wage increases. Difference-in-difference-in-difference models estimated the impact of minimum wage increases on hypertension among Black adults relative to White adults.

**Results:**

As state wage limits increase, the odds of hypertension significantly decreased among Black adults overall. This relationship is largely driven by the impact of these policies on Black women. However, the Black–White disparity in hypertension worsened as state minimum wage limits increased, and the magnitude of this disparity was larger among women.

**Conclusion:**

States having a minimum wage above the federal wage limit are not sufficient to combat structural racism and reduce the disparities in hypertension among Black adults. Rather, future research should explore livable wages as a policy lever to reduce disparities in hypertension among Black adults.

## Introduction

An estimated one in three U.S. adults are affected by hypertension.^1^ Non-Hispanic Black adults, in particular, are disproportionately burdened by earlier onset and higher prevalence of hypertension compared with other racial and ethnic groups.^2,3^ This disparity is largely attributed to racial and ethnic differences in socioeconomic status,^4^ which can impact health behaviors, access to care and other resources, and overall health and well-being.^5^ Indeed, lower income over the life course is associated with elevated risk of hypertension among Black adults.^6^ However, socioeconomic and health disparities among this population are not by happenstance, but rather the product of structural racism embedded in all facets of U.S. society.^7–9^

Decades of racist U.S. policy decisions have resulted in the over-representation of Black adults in lower wage occupations.^10^ Though minimum wage increases have been touted as a potential solution to economic and related health disparities,^11^ minimum wage laws have also been linked to structural racism^12^—the way in which society is structured to benefit White communities more than or at the expense of racially minoritized communities.^13^ Specifically, U.S. minimum wage limits were established in the Fair Labor Standards Act (FLSA) of 1938^14^ but excluded occupations largely held by Black workers, such as agricultural, domestic, and service workers.^15^

In addition, FLSA grants states the authority to increase minimum wages above the federal limit—31 states have exercised this authority^16^—but most states with a large Black population^17^ have not.^16^ Moreover, many states have enacted pre-emption laws that prohibit local (often urban) governments from raising minimum wage limits in their jurisdictions.^18^ Problematically, these discriminatory wage policies can have far-reaching and long-lasting negative implications for economic growth, upward mobility, and health-related outcomes among Black workers.

Though minimum wage increases have the potential to improve socioeconomic status,^19^ reduce psychosocial stress,^20^ increase access to health-related resources,^21,22^ and improve overall health outcomes,^22,23^ the evidence on the impact of minimum wage increases on cardiovascular health outcomes is inconsistent. For instance, states with minimum wages above the federal limit have lower rates of cardiovascular disease mortality than other states.^24^ However, minimum wage increases have been linked to increases in some cardiovascular risk factors, such as elevated body mass index, poor dietary quality, tobacco use, and obesity.^23,25–27^

Evidence also suggests that the effectiveness of minimum wage increases varies by race, ethnicity, and gender.^25^ Among Black adults, for example, minimum wage increases may have no significant impact on health^23^ or, in some cases, a negative impact on health^25^ compared with their White counterparts. This disparity can be explained by Assari's (2018) theory of “diminished gain” among Black Americans.^28^

According to this theoretical framework, the health benefits of socioeconomic resources are systematically smaller for Black adults, and this “diminished gain,” a consequence of structural racism, is the primary driver of health disparities among this group.^28^ Therefore, it is possible that minimum wage increases may have no impact or even exacerbate Black–White disparities in cardiovascular outcomes such as hypertension.

This study examines the relationship between state-level minimum wage policies and disparities in hypertension outcomes among Black and White adults. Specifically, our aims are to determine (1) whether state minimum wage increases are associated with decreased likelihood of hypertension among Black adults compared with Black adults in states without an increase and (2) whether minimum wage increases are associated with a decrease in the Black–White disparity over time. This contribution is particularly timely and relevant given increased public interest in racial health and wealth disparities. Our findings have implications for minimum wage policy and cardiovascular health equity.

## Methods

We used a quasi-experimental design to measure the impact of state-level minimum wage increases on Black–White disparities in hypertension among lower income adults. Because we used de-identified publicly available data, this study was deemed not human subjects research, and institutional review board approval was not required.

### Data

We used minimum wage data from the U.S. Department of Labor (DOL) from 2000 to 2019. These data include annual state-level minimum wages for nonfarm and nontipped employees, allowing us to track changes to state minimum wages over time. These data were merged with pooled cross-sectional survey data (2001–2019) from the Behavioral Risk Factor Surveillance System (BRFSS). BRFSS conducts annual phone surveys of a nationally representative sample of noninstitutionalized U.S. adults to collect data on health behaviors, chronic disease outcomes, and health care utilization.

Approximately 400,000 surveys are collected annually and, consequently, BRFSS is a critical epidemiological tool that has been widely used to assess hypertension trends and outcomes.^1,29–31^ In odd survey years, BRFSS respondents are asked hypertension-related questions, such as whether they have ever received a hypertension diagnosis. BRFSS data also include information on employment status and income.

### Sample

We limited our sample to respondents who self-identified as “Black” or “African American” or “White” but not of Hispanic, Latinx, or Spanish origin. Because Black adults experience earlier onset of hypertension,^32,33^ our sample includes adults of ages 25 years and older. Pregnant women, respondents not employed for wages, and those with missing data were excluded. Based on these criteria, the final analytic sample included 969,783 respondents.

### Outcome

Our outcome was a binary indicator of self-reported hypertension diagnosis based on the question “Have you ever been told by a doctor, nurse, or other health professional that you have high blood pressure?” Respondents who answered affirmatively were coded as “1” and all others were coded as “0.” We assessed this outcome among Black and White adults independently, and then compared the outcome between the two racial groups.

### Independent variable

State minimum wage limits are often adjusted incrementally over time. Therefore, our primary determinant of interest was a continuous state-year variable of the minimum wage limit. Five jurisdictions (Alabama, Louisiana, Mississippi, South Carolina, and Tennessee) have no minimum wage statute. In addition, codified minimum wage limits are set below the federal minimum wage in two jurisdictions—Georgia and Wyoming. Because the federal minimum pre-empts state minimums codified at or below the federal level, we used the federal-year minimum wage limit for these seven jurisdictions.

States with a minimum wage above the federal limit were considered our treatment group. Because minimum wage increases <5% can have unintended consequences,^34^ we coded a secondary continuous determinant as the difference between the state-year minimum wage minus the federal-year minimum wage.

### Covariates

Models adjusted for individual characteristics: age group, sex, educational attainment, and household (HH) size. Age group was categorized as early (ages 25–39 years), middle (ages 40–64 years), or late (ages ≥65 years) adulthood. Sex was dichotomized as female or male. Educational attainment was coded into four categories: no high school (HS) diploma, HS diploma or GED, some college, and college graduate. HH size was the total number of adults and children in the HH. Our models also adjust for time-varying contextual factors that are associated with the outcome of interest, such as the Great Recession,^35,36^ the enactment of the Affordable Care,^37^ and Medicaid expansion status.^38,39^

### Statistical analysis

Descriptive statistics were used to characterize the study sample and compare respondent characteristics in states with and without a minimum wage above the federal limit. We employed two modeling strategies to assess our outcome of interest. First, generalized difference-in-difference (DD)^40^ logistic regression models compared changes in the odds of hypertension after state minimum wage increases among Black and White adults separately. Unlike traditional DD models, generalized DD models account for staggered exposure to policy changes across states.^40^ The empirical model is expressed in the following equation:


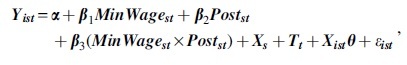


where $Y_{ist}$ is the probability of self-reported hypertension for individual *i* in state *s* in year *t*. $MinWage_{st}$ is a continuous measure of the minimum wage limit in state *s* after an increase above the federal wage limit in a given year *t*. $Post_{st}$ is an indicator of whether the state *s* observation year *t* is after the minimum wage increased above the federal wage limit. $MinWage_{st}\times Post_{st}$, the DD estimator, indicates whether the observation occurred in a treatment state after the minimum wage increased above the federal wage limit. *X_s_* and *T_t_* represent state and time fixed effects, respectively. $X_{st}\theta$ is a vector for all individual and state control variables, and $\delta_{ist}$ is the error term.

Next, we estimated the following generalized difference-in-difference-in-differences (DDD) logistic regression models to determine whether the odds of hypertension increased or decreased among Black adults compared with White adults after state-level minimum wage increases:


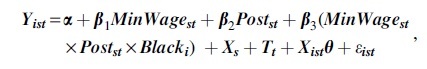


where the DDD estimator, $\left(MinWage_{st}\times Post_{st}\times Black_{i}\right)$, tests the interaction between the increased minimum wage and race to measure Black–White disparities in the outcomes. Finally, we repeated both models using a continuous measure of the calculated difference between state minimum wage and the federal minimum wage in a given year.

Black women are disproportionately burdened by poverty,^41^ so we repeated our models but stratified by sex to compare outcomes between Black and White women as well as Black men and White men. All models incorporated BRFSS survey design weights and used clustered robust standard errors. These analyses were performed using STATA 17 statistical software (StataCorp. 2021; Stata Statistical Software: Release 17. College Station, TX: StataCorp LLC.) and statistical significance was considered at the *p*<0.05 level.

## Results

**FIG. 1.**
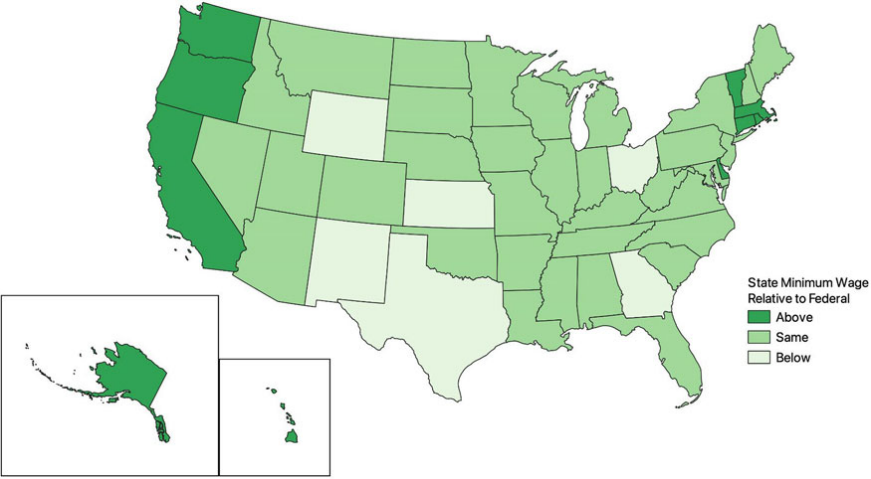
States with minimum wage limits above, at, or below the federal wage limit of $5.15 in 2001.

**FIG. 2.**
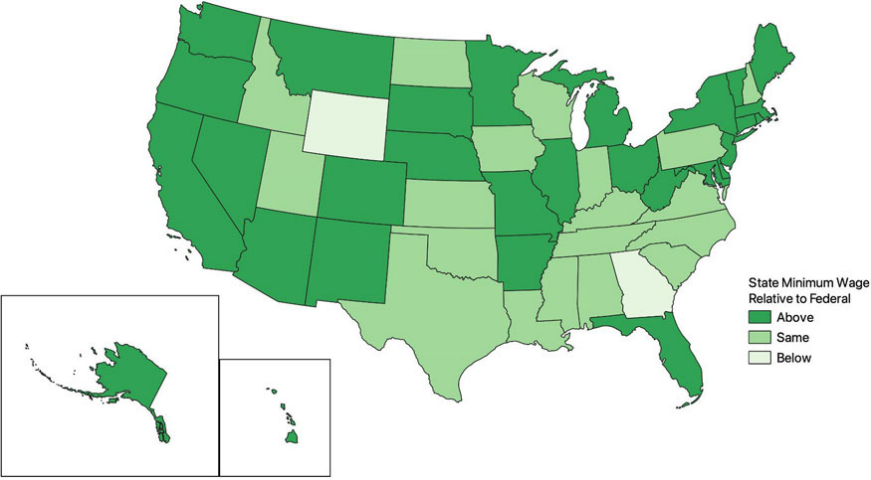
States with minimum wage limits above, at, or below the federal wage limit of $7.25 in 2019.

**FIG. 3.**
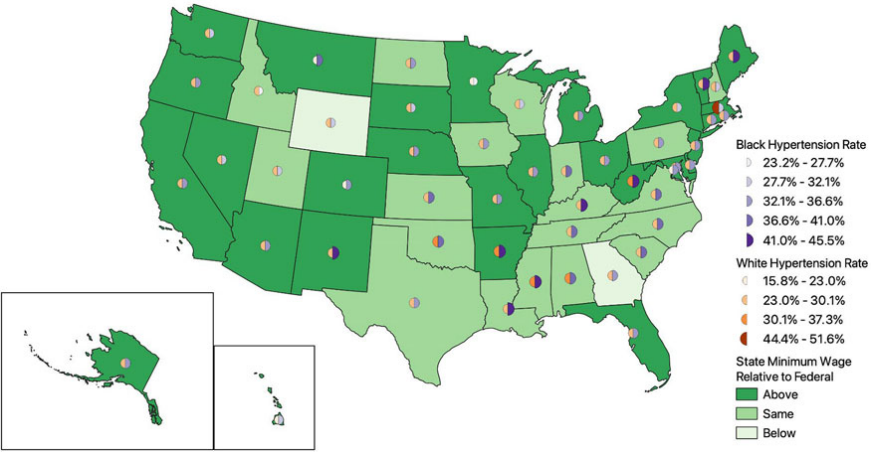
States with minimum wage limits above, at, or below the federal wage limit of $7.25 in 2019, with hypertension rates for *Black* and *White* adults in the study sample.

States' responses to federal minimum wage guidelines have evolved over time. From 2001 to 2019, there were multiple statutes setting the state-level minimum wage above the federal wage limit. Figures 1 and 2 map state-level minimum wages relative to federal wage limits in 2001 and 2019, respectively. Figure 3 displays the weighted proportion of Black and White adults in our study sample with hypertension. In 2001, ∼20% of jurisdictions had enacted a statute setting the minimum wage in their jurisdiction above the federal wage limit of $5.15 per hour (Fig. 1).

This percentage nearly tripled by 2019 with ∼60% of jurisdictions setting the minimum wage above the $7.25 per hour federal wage limit (Fig. 2). Notably, we observed that lower rates of hypertension did not always correlate with states having a minimum wage above the federal limit (Fig. 3).

Our unweighted sample included 83,301 non-Hispanic Black adults and 886,482 non-Hispanic White adults in states with (treatment) and without (control) minimum wages above the federal limit (Table 1). Among Black adults, the percentage with hypertension was slightly lower in control states (35.9% vs. 36.7%). The distribution of all other individual characteristics was mostly similar in treatment and control states. Among White adults, the distribution of individual characteristics was similar between treatment and control states; however, unlike Black adults, the percentage of White adults with hypertension was higher in treatment states relative to control states (28.2% vs. 25.5%).

**Table 1.** Weighted Descriptive Characteristics of Black and White Adults in States with (Treatment) and Without (Control) Minimum Wage Increases Above the Federal Wage Limit, 2001–2019

	Black adults		White adults	
	Unweighted *n*=83,301 Weighted *n*=10,741,637		Unweighted *n*=886,482 Weighted *n*=102,900,506	
	Treatment	Control	Treatment	Control
Hypertension diagnosis				
Yes	35.9.%	36.7%	28.2%	25.5%
Sex				
Female	60.8%	63.9%	52.4%	53.8%
Male	39.2%	36.1%	47.6%	46.2%
Age group				
Early (ages 25–39 years)	33.5%	36.9%	25.1%	29.9%
Middle (ages 40–64 years)	62.3%	59.9%	65.5%	64.9%
Late (ages ≥65 years)	4.2%	3.2%	9.4%	5.2%
Education				
Less than HS	4.0%	6.4%	2.0%	3.2%
HS diploma or GED	23.6%	30.4%	20.2%	25.8%
Some college	31.8%	29.6%	28.3%	27.1%
College graduate	40.5%	33.6%	49.5%	43.8%
HH size				
Mean (SE)	3.04 (0.02)	3.08 (0.01)	2.86 (0.06)	2.95 (0.003)

GED, general educational development; HH, household; HS, high school; SE, standard error.

In unadjusted models, state minimum wage increases were associated with a lower but not significant odds of hypertension among Black adults (odds ratio [OR]=0.98, 95% confidence interval [CI]=[0.95, 1.00]) (Table 2). However, this relationship was statistically significant in the fully adjusted model (OR=0.97, 95% CI=[0.95, 0.997]). By contrast, among White adults residing in treatment states, the odds of hypertension was higher in both unadjusted (OR=1.06, 95% CI=[0.95, 1.19]) and fully adjusted (OR=1.02, 95% CI=[0.97, 1.07]) models, but neither relationship was statistically significant. Lastly, we observed no statistically significant relationship between increased state–federal wage differences and the odds of hypertension among Black adults (OR=0.99, 95% CI=[0.95, 1.04]) or White adults (OR=1.09, 95% CI=[0.95, 1.25]).

**Table 2.** Estimated Effects of State Minimum Wage Increases on Hypertension Among Black and White Adults, 2001–2019

Predicted probability of having hypertension (OR [95% CI])				
	Black adults		White adults	
	Unadjusted OR [LL, UL]	Fully adjusted OR [LL, UL]	Unadjusted OR [LL, UL]	Fully adjusted OR [LL, UL]
DD estimator, $MinWage_{st}\times Post_{st}$ (continuous wage)	0.98 [0.95, 1.00]	0.97^a^ [0.95, 0.997]	1.06 [0.95, 1.19]	1.02 [0.97, 1.07]
Gender (ref: male)	—		—	
Female		1.03 [0.97, 1.10]		0.64^b^ [0.60, 0.68]
Age group (ref: early, 25–39 years)	—		—	
Middle (ages 40–64 years)		3.71^b^ [3.49, 3.95]		3.09^b^ [3.02, 3.17]
Late (ages ≥65 years)		8.66^b^ [7.32, 10.24]		8.31^b^ [6.40, 10.80]
Education (ref: <HS)	—		—	
HS diploma or GED		0.90 [0.80, 1.01]		0.90^b^ [0.85, 0.95]
Some college		0.88^a^ [0.78, 0.99]		0.88^b^ [0.80, 0.97]
College graduate		0.67^b^ [0.60, 0.74]		0.61^b^ [0.57, 0.65]
HH size	—	0.95^b^ [0.93, 0.96]	—	0.87^b^ [0.86, 0.89]
DD estimator, $MinWage_{st}\times Post_{st}$ (Difference between state and federal wage)	1.00 [0.96, 1.05]	0.99 [0.95, 1.04]	1.21 [0.91, 1.60]	1.09 [0.95, 1.25]

Notes: ^a^*p*<0.05; ^b^*p*≤0.01.

CI, confidence interval; DD, difference-in-difference; LL, lower limit; OR, odds ratio; UL, upper limit.

Table 3 displays the results from our generalized triple difference (DDD) models. Unadjusted results indicate that state minimum wage increases significantly increase the odds of hypertension among Black adults compared with White adults (OR=1.05, 95% CI=[1.03, 1.06]). This statistical relationship held in our fully adjusted model (OR=1.07, 95% CI=[1.06, 1.08]). When we repeated the model using our secondary determinant, the state–federal wage difference, the magnitude of the Black–White disparity in hypertension was much greater than we observed in our primary DDD model (OR=1.25, 95% CI=[1.18, 1.34]).

**Table 3.** Estimated Effects of State Minimum Wage Increases on Hypertension Among Black Compared with White Adults, 2001–2019

Predicted probability of having hypertension (OR [95% CI])		
	Unadjusted OR [LL, UL]	Fully adjusted OR [LL, UL]
DDD estimator, *MinWag_st_×Post_st_×Black*(continuous wage)	1.05^b^ [1.03, 1.06]	1.07^b^ [1.06, 1.08]
Gender (ref: male)		
Female	—	0.68^b^ [0.64, 0.73]
Age group (ref: early, 25–39 years)	—	
Middle (ages 40–64 years)		3.10^b^ [3.03, 3.18]
Late (ages ≥65 years)		8.28^b^ [6.29, 10.91]
Education (ref: <HS)	—	
HS diploma or GED		0.87^b^ [0.83, 0.92]
Some college		0.85^b^ [0.78, 0.93]
College graduate		0.59^b^ [0.56, 0.62]
HH size	—	0.81^b^ [0.75, 0.88]
DDD estimator, *MinWag_st_×Post_st_×Black* (difference between state and federal wages)	1.17^b^ [1.07, 1.26]	1.25^b^ [1.18, 1.34]

Notes: ^a^*p*<0.05; ^b^*p*≤0.01.

DDD, difference-in-difference-in-differences.

Table 4 displays the results of our supplemental analysis. As the state minimum wage increased, Black women residing in treatment states had a significantly lower odds of hypertension than Black women in control states (OR=0.97, 95% CI=[0.94, 0.99]). The relationship between state–federal wage difference and the odds of hypertension was not significant (OR=0.97, 95% CI=[0.93, 1.02]). Among White women, there was no statistically significant relationship between state minimum wage increases and hypertension.

**Table 4.** Estimated Effects of State Minimum Wage Increases on Hypertension Among Black and White Adults, Stratified by Sex, 2001–2019

Predicted probability of having hypertension (OR [95% CI])				
	Female adults		Male adults	
	Black, fully adjusted OR [LL, UL]	White, fully adjusted OR [LL, UL]	Black, fully adjusted OR [LL, UL]	White, fully adjusted OR [LL, UL]
DD estimator, $MinWage_{st}\times Post_{st}$ (continuous wage)	0.97^b^ [0.94, 0.99]	1.00 [0.98, 1.01]	0.99 [0.95, 1.02]	1.03 [0.96, 1.10]
DD estimator, $MinWage_{st}\times Post_{st}$ (difference between state and federal wages)	0.97 [0.93, 1.02]	1.01 [0.98, 1.03]	1.02 [0.95, 1.09]	1.11 [0.93, 1.31]
DDD estimator, $MinWage_{st}\times Post_{st}\times Black$ (continuous wage)	1.10^b^ [1.09, 1.11]	—	1.03^b^ [1.02, 1.05]	—
DDD estimator, $MinWage_{st}\times Post_{st}\times Black$ (difference between state and federal wages)	1.40^b^ [1.30, 1.50]	—	1.12^b^ [1.05, 1.18]	—

Notes: ^a^*p*<0.05; b*p*≤0.01.

In both DDD models, we observed a significantly higher odds of hypertension among Black women than among White women. State minimum wage increases were not associated with the odds of hypertension among Black or White men. However, DDD models suggest that state minimum wage increases are associated with an increased Black–White disparity in hypertension among men.

## Discussion

Higher income is a primary mechanism by which individuals achieve social mobility,^42^ reduce psychosocial stress,^20^ and improve related health outcomes.^42,43^ As such, advocates suggest that minimum wage policies present a salient opportunity for policymakers to make a meaningful impact on population health and health equity. However, the relationship between income and population health, especially cardiovascular health, is complex.^21^ Our study contributes to the empirical evidence base by applying a quasi-experimental design to a nationally representative sample of U.S. adults to determine whether minimum wage increases impact a leading cause of morbidity and mortality among non-Hispanic Black adults—hypertension.

Moreover, our study rigorously assesses the complex relationship between minimum wage increases and Black–White disparities in hypertension while highlighting the influence of structural racism.

Although most states have increased the minimum wage above the federal wage limit, our findings suggest that these increases do not ensure significant reductions in hypertension among Black adults. Though we observed an inverse relationship between state minimum wage increases and the odds of hypertension among Black adults in treatment states, this overall reduction can be attributed to changes among Black women. We did not observe a similar relationship among Black men. This could be attributed to Black women's over-representation in low-wage employment^10,44^ and higher likelihood of experiencing poverty,^41^ making this demographic more sensitive to mandated wage increases.

Problematically, Black–White disparities in hypertension appear to significantly worsen after states minimum wage increases. These findings are consistent with Assari's theory of “diminished gain” of health benefits from socioeconomic resources among Black adults compared with their White counterparts.^28,45^ In this case, state minimum wage increases disproportionately benefit White adults compared with their Black counterparts, thus exacerbating disparities in hypertension.

It is also likely that minimum wage increases alone cannot ameliorate the health consequences of structural racism because the policies themselves are a product of structural racism. Interestingly, we found that the magnitude of the disparity was smaller among men than we observed among women. This confirms that Black women bear the brunt of the disparate burden of hypertension on Black adults,^46^ highlighting the importance of assessing cardiovascular health disparities through an intersectional lens.

Alternatively, unintended consequences of minimum wage increases may negate their effectiveness as an antipoverty policy solution,^47^ especially among vulnerable populations. For example, social safety net eligibility is means-tested based on HH income.^48^ A larger proportion of Black HHs rely on these social safety net benefits compared with White HHs.^49,50^ Therefore, it is likely that Black HHs are disproportionately impacted by incremental minimum wage increases that render HHs ineligible for safety net benefits without sufficiently replacing the financial value of those benefits.

Similarly, minimum wage increases have been linked to negative outcomes in the labor market.^22,51–54^ Because Black workers often face significant discrimination in the labor market^55^ and are more likely to be negatively affected by adverse shocks to the labor market,^56^ it is likely that any unintended consequences of minimum wage increases on the labor market will disproportionately impact this group.

Importantly, Black adults will continue to face “diminished gain” of potential cardiovascular benefits of state-mandated wage increases because structural racism continues to shape this population's exposure to cardiovascular risk factors. Black families tend to reside in neighborhoods with unequal access to healthy foods, limited access to quality health care, and increased exposure to psychosocial stressors^57,58^; all of which can be linked to structural racism.^57,58^ Therefore, addressing the disparate impact of hypertension on Black adults will require the implementation of “a large-scale antiracist policy intervention,”^59^ such as a livable wage (an income that covers all HH needs) to combat structural racism, improve socioeconomic position, and reduce racial disparities in hypertension.

## Limitations

Results of this study are subject to several limitations. First, BRFSS only includes the hypertension-related questions in an optional module collected in odd survey years. Next, BRFSS data are cross-sectional, limiting our ability to infer causality. In addition, BRFSS does not include data on respondents' occupation, receipt of safety net benefits, or whether they are employed for hourly or salary wages, which would have enabled more precise exclusion criteria for our study sample. Also, minimum wage limits can be legislated at the local level if states do not have a pre-emption statute; however, city-level geographic identifiers are not included in the BRFSS data used.

Moreover, BRFSS does not identify respondents who reside in one state but are employed in another and, thus, subject to that state's wage policy. Also, our outcome of interest was based on the question, “Have you ever been told by a doctor, nurse, or other health professional that you have high blood pressure?” meaning our study sample is limited to adults with some level of access to care. Therefore, our findings may not be generalizable to adults with undiagnosed hypertension. Lastly, minimum wage policies have been linked to spillover effects on groups that should be unaffected.^51,60^ These data did not allow for the identification of a true placebo group or robust falsification testing.

## Conclusion

Over the past two decades, several U.S. states have steadily increased the minimum wage in their jurisdiction. However, these policies do not address the underlying driver of racial disparities in hypertension—structural racism. Future policy decisions should focus on the establishment of a livable wage as a policy lever to improve socioeconomic position among racially minoritized populations, reduce racial disparities in hypertension, and advance cardiovascular health equity.

## Abbreviations Used

BRFSS: Behavioral Risk Factor Surveillance System
CI: confidence interval
DD: difference-in-difference
DDD: difference-in-difference-in-differences
DOL: Department of Labor
FLSA: Fair Labor Standards Act
GED: general educational development
HH: household
HS: high school
LL: lower limit
OR: odds ratio
SE: standard error
UL: upper limit

## References

[1] Samanic CM, Barbour KE, Liu Y, et al. Prevalence of self-reported hypertension and antihypertensive medication use among adults—United States, 2017. Morb Mortal Wkly Rep 2020;69(14):393; doi: 10.15585/MMWR.MM6914A1 PMC7147902 32271727

[2] Carson AP, Howard G, Burke GL, et al. Ethnic differences in hypertension incidence among middle-aged and older adults: The multi-ethnic study of atherosclerosis. Hypertension 2011;57(6):1101–1107; doi: 10.1161/HYPERTENSIONAHA.110.168005 21502561 PMC3106342

[3] Virani SS, Alonso A, Aparicio HJ, et al. Heart disease and stroke statistics—2021 update. Circulation 2021;143:E254–E743; doi: 10.1161/CIR.0000000000000950 33501848 PMC13036842

[4] Kibria GM Al, Crispen R, Chowdhury MAB, et al. Disparities in absolute cardiovascular risk, metabolic syndrome, hypertension, and other risk factors by income within racial/ethnic groups among middle-aged and older US people. J Human Hypertens 2021;1–11; doi: 10.1038/s41371-021-00513-8 PMC8915051 33674704

[5] Dubay LC, Lebrun LA. Health, behavior, and health care disparities: Disentangling the effects of income and race in the United States. Int J Health Serv 2012;42(4):607–625; doi: 10.2190/HS.42.4.c 23367796

[6] Glover LM, Cain-Shields LR, Wyatt SB, et al. Life course socioeconomic status and hypertension in African American adults: The Jackson heart study. Am J Hypertens 2020;33(1):84–91; doi: 10.1093/AJH/HPZ133 31420642 PMC6931894

[7] Yearby R. Structural racism and health disparities: Reconfiguring the social determinants of health framework to include the root cause. J Law Med Ethics 2020;48(3):518–526; doi: 10.1177/1073110520958876. 33021164

[8] Bailey ZD, Krieger N, Agénor M, et al. Structural racism and health inequities in the USA: Evidence and interventions. Lancet 2017;389(10077):1453–1463; doi: 10.1016/S0140-6736(17)30569-X. 28402827

[9] Williams DR. Race and health: Basic questions, emerging directions. Ann Epidemiol 1997;7(5):322–333; doi: 10.1016/S1047-2797(97)00051-3 9250627

[10] Doede MS. Black jobs matter: Racial inequalities in conditions of employment and subsequent health outcomes. Public Health Nurs 2016;33(2):151–158; doi: 10.1111/phn.12241 26559050

[11] Derenoncourt E, Montialoux C. Minimum wages and racial inequality. Q J Econ 2020;136(1):169–228; doi: 10.1093/QJE/QJAA031

[12] Agénor M, Perkins C, Stamoulis C, et al. Developing a database of structural racism-related state laws for health equity research and practice in the United States. Public Health Rep 2021;136(4):428–440. 33617383 10.1177/0033354920984168 PMC8203034

[13] Gee GC, Hicken MT. Structural racism: The rules and relations of inequity. Ethn Dis 2021;31(Suppl. 1):293; doi: 10.18865/ED.31.S1.293 34045831 PMC8143846

[14] 75th United States Congress. Fair Labor Standards Act of 1938; 1938.

[15] Perea JF. The echoes of slavery: Recognizing the racist origins of the agricultural and domestic worker exclusion from the National Labor Relations Act. Ohio State Law J 2011;72.

[16] U.S. Department of Labor. Consolidated Minimum Wage Table. 2022. Available from: https://www.dol.gov/agencies/whd/mw-consolidated [Last accessed: November 3, 2022].

[17] Frey WH. Six Maps That Reveal America's Expanding Racial Diversity. 2019. Available from: https://www.brookings.edu/research/americas-racial-diversity-in-six-maps/ [Last accessed: November 3, 2023].

[18] Melton-Fant C. New preemption as a tool of structural racism: Implications for racial health inequities. J Law Med Ethics 2022;50(1):15–22; doi: 10.1017/JME.2022.4 35244004

[19] Cooper D. Raising the Minimum Wage to $12 by 2020 Would Lift Wages for 35 Million American Workers. Washington, DC; 2015. Available from: https://www.epi.org/publication/raising-the-minimum-wage-to-12-by-2020-would-lift-wages-for-35-million-american-workers/ [Last accessed: March 11, 2022].

[20] Svalestuen S. Is the mediating effect of psychosocial stress on the income–health relationship moderated by income inequality? SSM Popul Health 2022;20:101302; doi: 10.1016/J.SSMPH.2022.101302 36479320 PMC9720100

[21] Paul Leigh J, Leigh WA, Du J. Minimum wages and public health: A literature review. Prev Med (Baltim) 2019;118:122–134; doi: 10.1016/J.YPMED.2018.10.005 30316876

[22] Leigh JP, Du J. Effects of minimum wages on population health. Health Aff 2018; doi: 10.1377/HPB20180622.107025

[23] Andreyeva E, Ukert B. The impact of the minimum wage on health. Int J Health Econ Manag 2018;18(4):337–375; doi: 10.1007/S10754-018-9237-0/TABLES/23 29516331

[24] Van Dyke ME, Komro KA, Shah MP, et al. State-level minimum wage and heart disease death rates in the United States, 1980–2015: A novel application of marginal structural modeling. Prev Med (Baltim) 2018;112:97–103; doi: 10.1016/J.YPMED.2018.04.009 PMC5970990 29625130

[25] Narain KDC, Zimmerman FJ. Examining the association of changes in minimum wage with health across race/ethnicity and gender in the United States. BMC Public Health 2019;19(1):1–20; doi: 10.1186/S12889-019-7376-Y/TABLES/15 31395043 PMC6686560

[26] Buszkiewicz JH, Hill HD, Otten JJ. Association of state minimum wage rates and health in working-age adults using the National Health Interview Survey. Am J Epidemiol 2021;190(1):21–30; doi: 10.1093/AJE/KWAA018 32037444 PMC7946793

[27] Huang C, Liu F, You S. The impact of minimum wage increases on cigarette smoking. Health Econ 2021;30(9):2063–2091; doi: 10.1002/HEC.4362 34060694

[28] Assari S. Health disparities due to diminished return among Black Americans: Public policy solutions. Soc Issues Policy Rev 2018;12(1):112–145; doi: 10.1111/sipr.12042

[29] Fang J, Gillespie C, Ayala C, et al. Prevalence of self-reported hypertension and antihypertensive medication use among adults aged ≥18 years—United States, 2011–2015. Morb Mortal Wkly Rep 2018;67(7):219; doi: 10.15585/MMWR.MM6707A4 PMC5858041 29470459

[30] Park S, Gillespie C, Baumgardner J, et al. Modeled state-level estimates of hypertension prevalence and undiagnosed hypertension among US adults during 2013–2015. J Clin Hypertens 2018;20(10):1395–1410; doi: 10.1111/JCH.13388 PMC8031025 30251346

[31] Kherallah R, Al Rifai M, Kamat I, et al. Prevalence and predictors of cost-related medication nonadherence in individuals with cardiovascular disease: Results from the Behavioral Risk Factor Surveillance System (BRFSS) survey. Prev Med (Baltim) 2021;153:106715; doi: 10.1016/J.YPMED.2021.106715 PMC9125503 34242664

[32] Howard G, Cushman M, Moy CS, et al. Association of clinical and social factors with excess hypertension risk in black compared with white US adults. JAMA 2018;320(13):1338–1348; doi: 10.1001/JAMA.2018.13467 30285178 PMC6233849

[33] Kramer MR, Valderrama AL, Casper ML. Decomposing black-white disparities in heart disease mortality in the United States, 1973–2010: An age-period-cohort analysis. Am J Epidemiol 2015;182(4):302–312; doi: 10.1093/aje/kwv050 26199382 PMC4528952

[34] Lopresti JW, Mumford KJ. Who benefits from a minimum wage increase? ILR Rev 2016;69(5):1171–1190; doi: 10.1177/0019793916653595

[35] Burgard SA, Ailshire JA, Kalousova L. The great recession and health: people, populations, and disparities. Ann Am Acad Pol Soc Sci 2013;650(1):194–213; doi: 10.1177/0002716213500212

[36] Patel N, Kalra R, Bhargava A, et al. Ideal cardiovascular health among American adults after the economic recession of 2008–2009: Insights from NHANES. Am J Med 2019;132(10):1182.e5–1190.e5; doi: 10.1016/J.AMJMED.2019.06.004 31278932 PMC7048007

[37] 111th United States Congress. Affordable Care Act. 2010. Available from: http://uscode.house.gov/view.xhtml?path=/prelim@title42/chapter157&edition=prelim [Last accessed: May 22, 2019].

[38] Cole MB, Kim J-H, Levengood TW, et al. Association of Medicaid expansion with 5-year changes in hypertension and diabetes outcomes at federally qualified health centers. JAMA Health Forum 2021;2(9):e212375; doi: 10.1001/JAMAHEALTHFORUM.2021.2375 35977186 PMC8796924

[39] Huguet N, Larson A, Angier H, et al. Rates of undiagnosed hypertension and diagnosed hypertension without anti-hypertensive medication following the affordable care act. Am J Hypertens 2021;34(9):989–998; doi: 10.1093/AJH/HPAB069 33929496 PMC8457435

[40] Richardson DB, Ye T, Tchetgen EJ. Generalized difference-in-differences. Epidemiology 2023;34(2):167–174; doi: 10.1097/EDE.0000000000001568 36722798

[41] Fins A. National Snapshot: Poverty among Women & Families, 2020. Washington, DC; 2020. Available from: https://nwlc.org/wp-content/uploads/2020/12/PovertySnapshot2020.pdf [Last accessed: June 26, 2022].

[42] Chetty R, Hendren N, Kline P, et al. Is the United States still a land of opportunity? Recent trends in intergenerational mobility. Am Econ Rev 2014;104(5):141–147; doi: 10.1257/AER.104.5.141

[43] Hyde S. Income and Health Outcomes. 2017. Available from: https://www.bls.gov/opub/mlr/2017/beyond-bls/income-and-health-outcomes.htm [Last accessed: November 17, 2022].

[44] Kim M. Women Paid Low Wages: Who They Are and Where They Work. 2000. Available from: https://www.bls.gov/opub/mlr/2000/article/women-paid-low-wages-who-they-are-and-where-they-work.htm [Last accessed: November 14, 2022].

[45] Assari S. Unequal gain of equal resources across racial groups. Int J Health Policy Manag 2018;7(1):1; doi: 10.15171/IJHPM.2017.90 29325397 PMC5745862

[46] Kalinowski J, Taylor JY, Spruill TM. Why are young black women at high risk for cardiovascular disease? Circulation 2019;139(8):1003–1004; doi: 10.1161/CIRCULATIONAHA.118.037689 30779648 PMC6383791

[47] Overstreet D. Is minimum wage an effective anti-poverty tool? J Poverty 2021;25(5):453–464; doi: 10.1080/10875549.2020.1869660

[48] Blavin F, Gangopadhyaya A. How the Minimum Wage Affects the Health Insurance Coverage, Safety Net Program Participation, and Health of Low-Wage Workers and Their Families: A Review of Recent Literature. Washington, DC; 2022. Available from: https://www.urban.org/research/publication/how-minimum-wage-affects-health-insurance-coverage-safety-net-program [Last accessed: November 14, 2022].

[49] U.S. Department of Agriculture F and NSO of PS. Characteristics of Supplemental Nutrition Assistance Program Households: Fiscal Year 2019. Alexandria, VA; 2021. Available from: https://www.fns.usda.gov/snap/characteristics-snap-households-fy-2019 [Last accessed: November 14, 2022].

[50] Medicaid and CHIP Payment and Access Commission (MACPAC). Report to Congress on Medicaid and CHIP Medicaid and CHIP Payment and Access Commission. Washington, DC; 2022. Available from: https://www.macpac.gov/wp-content/uploads/2022/06/MACPAC_June2022-WEB-Full-Booklet_FINAL-508-1.pdf [Last accessed: November 14, 2022].

[51] Gregory T, Zierahn U. When the minimum wage really bites hard: The negative spillover effect on high-skilled workers. J Public Econ 2022;206:104582; doi: 10.1016/J.JPUBECO.2021.104582

[52] Dube A, William Lester T, Reich M. Minimum wage shocks, employment flows, and labor market frictions. J Labor Econ 2016;34(3):663–704; doi: 10.1086/685449/SUPPL_FILE/12078DATA1.ZIP

[53] Royalty A. Do Minimum Wage Increases Lower the Probability That Low-Skilled Workers Will Receive Fringe Benefits? JCPR Working Papers. Northwestern University/University of Chicago Joint Center for Poverty Research; 2001.

[54] Marks MS. Minimum wages, employer-provided health insurance, and the non-discrimination law. Ind Relat J Econ Soc 2011;50(2):241–262; doi: 10.1111/J.1468-232X.2011.00635.X

[55] Wilson V Jr. WD. Understanding Black-White Disparities in Labor Market Outcomes Requires Models That Account for Persistent Discrimination and Unequal Bargaining Power. Economic Policy Institute: Washington, DC, USA; 2022.

[56] De K, Compton RA, Giedeman DC, et al. Macroeconomic shocks and racial labor market differences. South Econ J 2021;88(2):680–704; doi: 10.1002/SOEJ.12534

[57] Williams DR, Mohammed SA. Racism and health I: Pathways and scientific evidence. Am Behav Sci 2013;57(8):1152–1173; doi: 10.1177/0002764213487340 PMC3863357 24347666

[58] Williams DR, Mohammed SA. Racism and health II: A needed research agenda for effective interventions. Am Behav Sci 2013;57(8):1200–1226; doi: 10.1177/0002764213487341 PMC3863360 24347667

[59] McClendon J, Chang K, J. Boudreaux M, et al. Black-White racial health disparities in inflammation and physical health: Cumulative stress, social isolation, and health behaviors. Psychoneuroendocrinology 2021;131; doi: 10.1016/J.PSYNEUEN.2021.105251 34153589

[60] Autor DH, Manning A, Smith CL. The contribution of the minimum wage to U.S. wage inequality over three decades: A reassessment. Am Econ J Appl Econ 2016;8(1):58–99.

